# Promise of Systems Research in reducing burden of Mental Health

**DOI:** 10.1017/gmh.2014.3

**Published:** 2014-09-11

**Authors:** Abdul Ghaffar

It gives me great pleasure to participate in the launch of this inaugural issue of *Global Mental Health* as an Associate Editor for Policy and Systems. In my capacity as the Executive Director of the Alliance for Health Policy and Systems Research at WHO, I have been privileged to witness the expansion of Health Policy and Systems Research and its increasing relevance and impact in other fields, including mental health. Work in mental health globally is increasingly concerned with issues that are cross-cutting and inter-disciplinary in nature: the use of task shifting as a strategy for enabling access to care, the use of implementation research as a means of putting into practice knowledge about the interactions between sociocultural factors and mental health, as well as service delivery, financing, access to medicines, and other challenges related to the way that health systems are organized around the world.

In these and other areas, there are valuable lessons generated from the advancements in the field of mental health that can help inform our understanding of health systems as well as other areas of global health. But in turn, the field of mental health also has much to learn and take from the evolving field of health systems research. There is growing recognition that addressing the global burden of mental illness – which are among the leading causes of disability in all regions of the world, requires a strong focus on the design and capacity of health systems and policies that ultimately drive mental health care.

This natural convergence of priorities and goals offers a unique opportunity to bring mental health into the broader Health Policy and Systems Research agenda. Since the inception of the Alliance for Health Policy and Systems Research in 1999 and following the successes of the two Global Symposia on Health Systems Research in 2010 and 2012 as well as the launch of Health Systems Global, issues ranging from the use of econometrics in health systems planning to pathways for achieving universal health coverage have gained prominence in global health dialogues. What has been and is still missing from these discussions, however, is the attention to how these issues relate to mental health.

As the Associate Editor for Policy and Systems, I will work to encourage the use of Health Policy & Systems Research Methods and Approaches, as tools for addressing current mental health challenges. More importantly, we hope that this journal will serve as a platform to bring together the diverse range of actors and stakeholders whose collective actions are needed to respond to complex system challenges posed by the emerging burden of mental illness globally. The alignment of research agendas – along with the coordinated efforts of policy makers, practitioners, planners, and multi-disciplinary researchers, will be critical to the attainment of *Global Mental Health* goals.

We invite people from both communities of practice to see themselves as contributors to and users of the kind of research in this area we hope to publish, and to assist in forming a more effective and rich field of Health Policy and Systems Research as a result.

